# Identification of volatile and nonvolatile compounds in Citri Reticulatae Pericarpium Viride using GC–MS, UPLC‐Q‐Exactive Orbitrap‐MS, and HPLC‐PDA

**DOI:** 10.1002/fsn3.3181

**Published:** 2023-01-04

**Authors:** Jingxuan Li, Shiqi Zou, Wanling Yang, Mengdie Peng, Baizhong Chen, Jinji Deng, Minyan Wei, Guodong Zheng

**Affiliations:** ^1^ Guangzhou Municipal and Guangdong Provincial Key Laboratory of Molecular Target & Clinical Pharmacology, The NMPA and State Key Laboratory of Respiratory Disease, School of Pharmaceutical Sciences and the Fifth Affiliated Hospital Guangzhou Medical University Guangzhou China; ^2^ Guangdong Xinbaotang Biological Technology Co., Ltd Jiangmen China

**Keywords:** Citri Reticulatae Pericarpium Viride, GC–MS, HPLC–PDA, UHPLC‐Q‐Exactive orbitrap‐MS

## Abstract

Gas chromatograph–mass spectrometer (GC–MS), ultra‐high‐performance liquid chromatograph‐Q‐Exactive Orbitrap tandem mass spectrometry (UHPLC‐Q‐Exactive Orbitrap‐MS), and high‐performance liquid chromatography–photodiode array detection (HPLC–PDA) were used to qualitatively and quantitatively analyze the chemical component of Citri Reticulatae Pericarpium Viride “Geqingpi” (GQP). First of all, the volatile components of GQP are identified by GC–MS. Totally 56 volatile components were determined, and γ‐Terpinene (33.39%) and D‐Limonene (22.95%) were the main terpenes. Secondly, UHPLC‐Q‐Exactive Orbitrap‐MS was used for identifying nonvolatile compositions and 42 compositions were identified totally, including 23 flavonoids, nine organic acids, three coumarins, two alkaloids compounds, and five other compounds, among which nine of the determined constituents were detected for the first time in GQP. Thirdly, the content of seven main constituents in GQP was quantitatively analyzed via HPLC‐PDA, which were synephrine, hesperidin, limonin, nobiletin, HMF, tangeretin, and 5‐HPMF. Further investigation for quantitative analysis of seven bioactive compounds suggested that the concentration of hesperidin in GQP approximately was 16.0% (160.78 ± 0.95 mg·g^−1^), which was far higher than the standard for identification and quality control of CRPV in Chinese Pharmacopoeia (2020 edition) that “the content of hesperidin shall not be less than 5.0%.” The phytochemicals of GQP were elucidated in this study, which might be supporting information for identification between GQP and Citri Reticulatae Pericarpium Viride “Sihuaqingpi” (SHQP) and provided a scientific basis for the further active ingredient for pharmacological research and development prospects of GQP.

## Graphical abstract

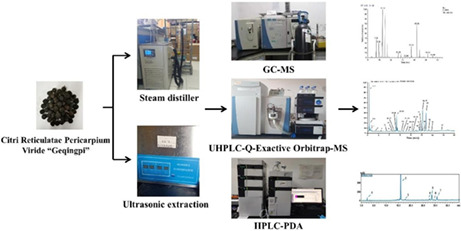

## 1 INTRODUCTION

Citri Reticulatae Pericarpium Viride (CRPV), which is called “Qing Pi” in Chinese, is derived from the dried infancy fruit or immature peel of *Citrus reticulata* Blanco and its cultivar. CRPV has two types, “Geqingpi” and “Sihuaqingpi”, on the basis of harvest time and method of processing (Pharmacopoeia, 2020). The dried fruitlet of *Citrus reticulata* Blanco that fall naturally is collected in May and June, called “Geqingpi” (GQP). GQP is often used for dyspepsia, expectorant, asthma, and liver. GQP has the characteristics of both medicine and food, for example, GQP, which is often used for tea and soup, is developed as a citrus fruit tea with the effect of regulating Qi, strengthening spleen, soothing liver, and moistening lung (Jiang & Miao, 2003). Special attention is paid to the fact that GQP is mainly used to break qi and resolve stagnation, while “Sihuaqingpi” is mainly used to regulate liver and regulate qi (Liu et al., 2021). The chemical constituents of the GQP are very rich, such as volatile oil, flavonoids, amino acids, and so on (Sheng et al., 2017).

As traditional Chinese medicine, Citri Reticulatae Pericarpium (CRP) is the dried mature pericarp of *Citrus reticulata* Blanco and its cultivars. There are amounts of researches focused on chemical component analysis for CRP (Mei et al., 2021; Zheng et al., 2021) whereas lacking systematical study on chemical profiling for CRPV. For instance, research concerning the analysis of characteristic volatile components on CRP at three maturity stages from July to December has been reported (Yi et al., 2015). However, there is no specific report on the volatile components of GQP collected in May and June. Regarding the nonvolatile component profiling of GQP, some studies have focused on flavonoids, such as hesperidin and polymethoxyflavones (PMFs) (e.g., nobiletin and Tangeretin) (Cheng et al., 2022; He et al., 2021). In brief, there is a lack of complete research reports on the chemical constituents of GQP.

It is necessary to combine various analytical techniques to analyze their phytochemical constituents (Wang et al., 2021; Zheng, Yang, et al., 2020), because of the complexity and variety of natural medicinal plants. The UHPLC‐Q‐Exactive Orbitrap‐MS technology is a useful analytical technology to satisfy these requirements, which integrates the separation ability of UHPLC with the qualitative function of mass spectrometry for compounds. It possesses the advantages of high resolution and high sensitivity, for which it can successfully separate complex components in natural products in a short time (Dong et al., 2021).

In order to rapidly and accurately identify chemical constituents of GQP, GC–MS, and UHPLC‐Q‐Exactive Orbitrap‐MS were, respectively, used as semiquantitative analysis for the volatile components and qualitative analysis for nonvolatile components. Furthermore, HPLC–PDA was used to quantitatively analyze the main active compositions in the nonvolatile components. The chemical composition of GQP was comprehensively identified in the present study. It might provide a detailed basis for quality control and further reasonable development and application of GQP.

## 2 MATERIALS AND METHODS

### 2.1 Materials and reagents

The premature fruitlet of *Citrus reticulata* Blanco was collected from Xinhui District, Jiangmen, Guangdong Province in May 2020. After naturally sun dried, GQP were obtained and were identified by Professor Guodong Zheng. After that, GQP were conserved at the Laboratory of Pharmacognosy, Guangzhou Medical University, Guangdong Province, China.

The reference standards (purity >98%) were purchased from Chengdu Must Biotechnology. They were hesperidin, nobiletin, synephrine, ferulic acid, limonin, and tangeretin. The other standards (purity >98%), stachydrine and 3,5,6,7,8,3′,4′‐heptamethoxyflavone, were purchased from Weikeqi Biotechnology and 5‐hydroxy‐6,7,8,3′,4′‐pentamethoxyflavone was purchased from Nanjing Chunqiu Biological Engineering. Ultrapure water was available using a Milli‐Q system. The Chromatographic‐grade acetonitrile, hexane, and methanol were obtained from Honeywell International Inc., and formic acid was obtained from Thermo Fisher Scientific.

### 2.2 Preparation of samples and standards

Geqingpi was cut into small pieces before the start of the experiment and pulverized into powder with an oscillating crusher. A total of 30‐g GQP were used for the extraction of volatile constituents based on the method described in 2020 edition of Chinese Pharmacopoeia (Li & Schluesener, 2017). After fully soaking with ultrapure water (300 ml) for 12 h and continuously heating for 5 h by a TC‐15 electric jacket (Xinhua Medical equipment Factory), volatile components were concentrated and returned to the volatile oil analyzer (Sichuan ShuBo (Group) Co. Ltd). A mixture of aliquot volatile compounds (50 μl) and hexane solvent (950 μl) was subjected to GC–MS analysis after filtration through a 0.22 μm filter. The content of volatile components (g·kg^−1^) = obtained volatile oil (g)/sample powder (kg), through which the content of volatile compounds is calculated.

Approximately 0.2 g GQP powder that was sieved through a 40‐mesh sieve was precisely weighed, and 20‐ml methanol was added to a KQ‐800KDE instrument (Kunshan Ultrasonic Instruments Co. Ltd.) for ultrasonic extraction (parameter: 320 W, 40 kHz) for 30 min. After filtration, the filtrates can be subjected to UHPLC‐Q‐Exactive Orbitrap‐MS analysis and HPLC–PDA analysis of seven bioactive components in GQP, taken through 0.22 μm PTFE microporous membrane filter. An appropriate amount of reference materials were weighed, dissolved in methanol, and then stored in a freezer at 4°C, which we use for the analysis by UHPLC‐Q‐Exactive Orbitrap‐MS. A methanol mixture of synephrine, limonin, 5‐HPMF, nobiletin, HMF, tangeretin, and hesperidin was diluted to different concentrations for establishing calibration curves, respectively, for HPLC–PDA analysis.

### 2.3 GC–MS analysis conditions

The gas chromatography (GC)–mass spectrometry (MS) system comprised an Agilent 7890A gas chromatograph system (Agilent) and a 5975C Triple‐Axis mass selective detector (Agilent). The chromatographic column was DB‐5MS ultra inert capillary column (30 m × 0.25 mm × 0.25 μm) with a good separation effect. Application settings were as below: the injection port temperature of 270°C, split ratio of 10:1, injection volume of 5 μl, carrier gas is high‐purity helium, and column flow of 1 ml· min^−1^. The initial temperature was 60°C, followed by a temperature increase to 80°C at a pace rate of 1°C· min^−1^ for 10 min, and then increased to 250 °C at a pace rate of 5 °C· min^−1^ and to 300°C at 20°C·min^−1^ finally, holding for 1 min. Mass spectrometry was conducted in full‐scan electron impact (EI+) mode at 70 eV to gain positive ions, scanning the range from m/z 30 to 550 amu (scan speed of 0.2 amu·s^−1^). The detector temperature is 270°C and the solvent delay time is 4 min. The mass spectrometry database is the NIST08.L database.

### 2.4 UHPLC‐Q‐Exactive Orbitrap‐MS system conditions

0.1% formic acid in water (phase A) and acetonitrile (phase B) make up the mobile phase. Using a ZORBAX Eclipse Plus C_18_ column (2.1 mm × 50 mm, 1.8 μm), the methanol extracts of GQP were separated at 40°C at the flow velocity of 0.4 ml·min^−1^. Gradient conditions were flowing: 0–2 min, 95% A; 2–4 min, 95%–75% A; 4–6 min, 75% A; 6–10 min, 75%–50% A; 10–14 min, 50%–15% A; 14–16 min, 15%–10% A. Equipped with the heated electrospray ionization (HES‐II) source, high‐resolution MS source parameters were operated in positive ion mode. Source parameters were as below: the ion spray voltage was set at 3.5 kV, the injection volume was 1 μl, and capillary temperature was 320°C. Sheath gas, auxiliary gas, and purge gas are all nitrogen and were sustained at 45.0, 15.0, and 3.0 L·min^−1^. The mass scanning range of full‐scan mode was *m/z* 70 ~ 1000 Da. All operations mentioned above were carried out in Xcalibur software, and there is no doubt that data processing was performed by Metworks software.

### 2.5 HPLC–PDA analysis conditions

For the analysis of the seven phytochemical compositions, SIL‐20A HPLC (Shimadzu) instrument was equipped with an SPDM20A detector (Shimadzu). The detection wavelengths were 209 nm (limonin), 224 nm (synephrine), 283 nm (hesperidin), and 330 nm (tangeretin, 5‐HPMF, nobiletin, and HMF). The standard solution of the above seven compounds was then diluted with methanol to appropriate concentrations to construct the calibration curves. At a temperature of 30°C with an injection volume of 10 μl and eluent flow rate of 1.0 ml·min^−1^, separations were conducted using a Diamonsil C_18_ column (250 mm × 4.6 mm, 5 μm). The mobile phase contained phosphoric acid water solution, pH = 3.70 (A) and methanol/acetonitrile (B, v/v = 1/1), with the following gradient variations: 0–5 min, 95% A; 5–10 min, 95%–45% A; 10–15 min, 45%–40% A; 15–20 min, 40%–35% A; 20–25 min, 35%–25% A; 25–30 min, 25%–15% A; 35–40 min, 15%–5% A. Linearity of the response, stability, precision, repeatability, and recovery verified by utilizing the HPLC–PDA method.

## 3 RESULTS

### 3.1 Determination of volatile constituents by GC–MS analysis

In the present study, 1.18% of volatile compounds were extracted from GQP. It was discovered that a total of 56 volatile constituents were identified in GQP, making up 99.93% of all identified volatile constituents. The corresponding relative contents are shown in Table 1, including 22 terpenes, 12 alcohols, 3 phenols, 2 ketones, 1 aldehyde, 5 esters, and 11 others. Terpenes were the prime volatile components, representing for relative content of 70%, of which mainly included γ‐Terpinene (33.39%) and D‐Limonene (22.95%).

**TABLE 1:** Fifty‐six volatile compounds identified in GQP by GC–MS

No.	t_R_ (min)	Compound formula	Identified compounds	Relative percentage (%)
Terpenes				
1	6.98	C_10_H_16_	4‐Methyl‐1‐(1‐methylethyl) bicyclo[3.1.0]hexane didehydro deriv	1.40
2	7.30	C_10_H_16_	α‐Pinene	4.46
3	7.99	C_10_H_16_	Camphene	0.08
4	9.09	C_10_H_16_	Sabinene	0.02
5	9.36	C_10_H_16_	β‐Pinene	4.02
6	10.00	C_10_H_16_	Myrcene	1.57
7	10.95	C_10_H_16_	α‐Phellandrene	0.20
8	11.63	C_10_H_16_	α‐terpinene	1.71
10	12.71	C_10_H_16_	D‐Limonene	22.95
12	14.92	C_10_H_16_	γ‐Terpinene	33.39
15	16.65	C_10_H_16_	Terpinolene	3.31
20	21.50	C_10_H_14_	1,3,8‐p‐Menthatriene	0.03
32	39.34	C_15_H_24_	δ‐Elemene	0.02
33	40.95	C_15_H_24_	Copaene	0.07
34	41.54	C_15_H_24_	β‐Elemene	0.03
36	42.59	C_15_H_24_	Caryophyllene	1.47
38	44.79	C_15_H_24_	Eremophilene	0.02
39	45.03	C_15_H_24_	α‐Serinene	0.18
40	45.37	C_15_H_24_	α‐Farnesene	0.41
41	45.70	C_15_H_24_	β‐Cadinene	0.12
44	47.43	C_15_H_24_O	Caryophyllene oxide	0.13
46	48.73	C_15_H_24_	β‐Guaiene	0.02
Alcohols				
17	17.99	C_10_H_18_O	Linalool	0.44
18	19.41	C_10_H_18_O	Fenchol	0.04
19	19.93	C_10_H_18_O	2‐Cyclohexen‐1‐ol, 1‐methyl‐4‐(1‐methylethyl)‐	0.02
21	24.92	C_10_H_18_O	Borneol	0.06
22	26.25	C_10_H_18_O	Terpinen‐4‐ol	1.42
23	27.24	C_10_H_14_O	2‐ (4‐Methylphenyl) propan‐2‐ol	0.08
24	28.49	C_10_H_18_O	α‐Terpineol	1.37
25	32.57	C_10_H_18_O	Nerol	0.02
42	46.54	C_15_H_26_O	Elemol	0.02
43	47.29	C_15_H_24_O	Spathulenol	0.05
47	49.01	C_15_H_26_O	T‐muurolol	0.02
48	49.30	C_15_H_26_O	α‐eudesmol	0.06
Aldehydes				
49	51.50	C_15_H_22_O	α‐Sinensal	0.41
Ketones				
27	34.65	C_10_H_16_O	Piperitone	0.02
45	48.20	C_20_H_28_O_2_	Metandienone	0.09
Phenols				
29	36.92	C_10_H_14_O	Carvacrol	0.35
30	37.43	C_10_H_14_O	Thymol	2.15
31	38.09	C_9_H_10_O_2_	2‐methoxy‐4‐vinylphenol	0.03
Esters				
35	42.24	C_9_H_11_NO_2_	Benzoic acid, 2‐(methylamino)‐, methyl ester	10.40
37	44.52	C_10_H_13_NO_2_	Benzoic acid, 2‐(methylamino)‐, ethyl ester	0.91
50	55.27	C_17_H_34_O_2_	Methyl palmitate	0.06
53	58.51	C_19_H_34_O_2_	Methyl linoleate	0.04
54	58.61	C_19_H_32_O_2_	Methyl linolenate	0.05
Others				
9	12.11	C_10_H_14_	p‐Cymene	5.37
11	13.74	C_10_H_16_	Ocimene Mixture of Isomers	0.14
13	15.03	C_7_H_9_N	N‐Methylaniline	0.05
14	15.59	C_8_H_16_	1‐cyclopropylpentane	0.02
16	16.97	C_10_H_12_	p‐cymene	0.13
26	33.06	C_11_H_16_O	2‐isopropyl‐4‐methylanisole	0.13
28	36.05	C_8_H_9_NO	2‐Methylformanilide	0.03
51	55.98	C_16_H_32_O_2_	Palmitic acid	0.21
52	56.60	C_22_H23N	4‐(1‐Methylethyl)‐N, α‐diphenyl benzenemethanamine	0.03
55	59.19	C_18_H_32_O_2_	Linoleic acid	0.04
56	59.29	C_18_H_30_O_2_	Linolenic acid	0.06

### 3.2 Identification of nonvolatile compounds by UHPLC‐Q‐Exactive Orbitrap‐MS

**FIGURE 1:**
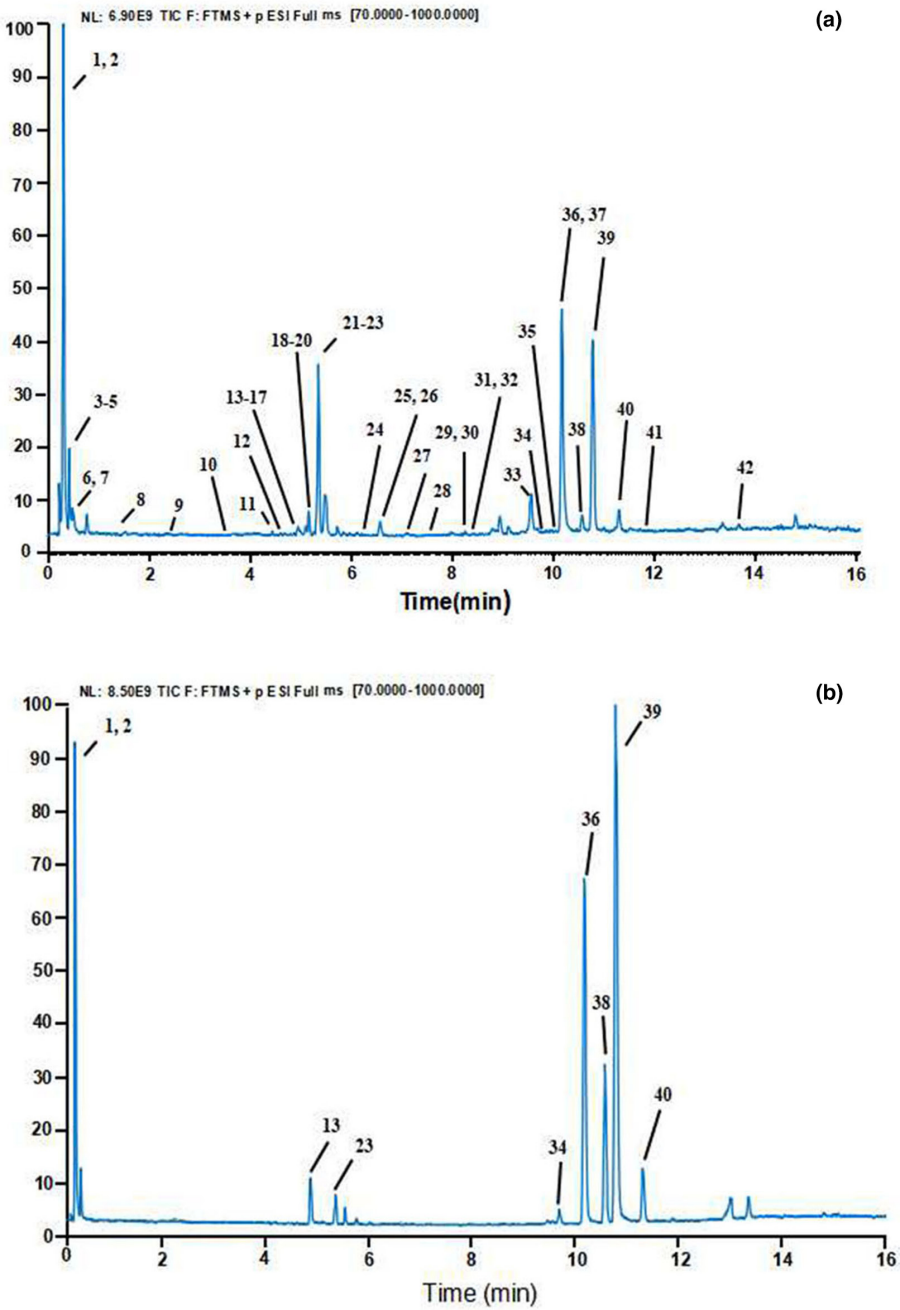
Total ion chromatograms (TIC) of GQP (a) and mixed standards (b)

Several parameters, such as the flow rate and elution gradient of the mobile phase, were optimized in this study, and the improved analytical conditions were obtained to qualitatively identify methanol extracts of GQP via UHPLC‐Q‐Exactive Orbitrap‐MS. Within 16 min, 42 constituents in total were successfully separated and detected in positive ion mode. It is clear that Figure 1 shows the total ion chromatogram (TIC) of GQP (A) and mixed reference standards (B). The mass spectrum fragmentation information and retention time were obtained in accordance with UHPLC‐Q‐Exactive Orbitrap‐MS and referring to Orbitrap Chinese Traditional Medicine Library (OCTML) and related literatures, totally 42 components were identified from GQP **(**Table 2 **)**, including 23 flavonoids (eight flavonoids glycosides, eight PMFs, and seven other flavonoids), nine organic acids, two alkaloids, three coumarins, and five other compounds. Nine of the determined constituents were reported for the first time in CRPV, containing two flavonoids, one organic acid, three coumarins, and three other compounds. These compounds were vitexin (Lin et al., 2020), iristectorigenin (Fu et al., 2020), isoferulic acid (Duan et al., 2014), esculin (Wang et al., 2016), esculetin (Li et al., 2012), 5,7‐Dimethoxycoumarin (Duan et al., 2014), 5‐Hydroxymethylfurfural (Zheng, Yang, et al., 2020), methyl ferulate, (Zheng, Yang, et al., 2020) and ethyl4’‐hydroxy‐3′‐methoxycinnamate (Zheng, Yang, et al., 2020).

**TABLE 2:** Forty‐two nonvolatile compounds identified in GQP by UHPLC‐Q‐Exactive orbitrap‐MS.

No	RT (min)	(M + H^+^) *m/z*	Major MS^2^ fragment ions (m/z)	Compound formula	Identification	Ref/standard
Flavonoid glycoside						
15	4.89	597.1815	435.1276, 399.1086, 355.0812, 301.0711, 289.0706, 263.0556, 219.0293, 195.0289, 163.0390, 153.0182, 129.0546, 85.0290, 71.0499	C_27_H_32_O_15_	Eriocitrin	15, b
16	4.93	433.1131	415.1016, 397.0918, 379.0812, 367.0808, 337.0706, 323.0921, 313.0706, 295.0616, 283.0602 267.0655, 183.0288, 165.0185, 121.0289, 91.0544	C_21_H_20_O_10_	Vitexin	13, b
17	4.94	595.1660	527.4942, 449.1079, 415.1025, 287.0550, 269.0442, 241.0499, 171.0286, 153.0183, 85.0290	C_27_H_30_O_15_	Kaempferol −3‐O‐rutinoside	Dou et al. (2017), b
18	5.16	581.1868	449.1209, 419.1331, 383.1124, 365.1015, 339.0860, 273.0757, 263.0550, 195.0288, 171.0289, 153.0183, 129.0547, 119.0494, 85.0290	C_27_H_32_O_14_	Naringin	15, b
20	5.18	579.1713	433.1130, 350.1698, 313.0671, 271.0601, 247.0599, 225.0544, 171.0289, 153.0183, 119.0494	C_27_H_30_O_14_	Rhoifolin	15, b
21	5.33	609.1815	463.1235, 301.0707, 286.0473, 258.0523, 171.0289, 129.0548, 85.0290	C_28_H_32_O_15_	Diosmin	15, b
23	5.36	611.1968	303.0864, 263.0551, 219.0288, 195.0290, 177.0547, 153.0183, 129.0548, 85.0290, 71.0499	C_28_H_34_O_15_	Hesperidin	15, a, b
26	6.57	595.2021	499.1456, 415.1376, 397.1280. 379.1158, 329.1012, 287.0914, 263.0551, 219.0286, 195.0289, 171.0288, 161.0598, 153.0183, 85.0290	C_28_H_34_O_14_	Poncirin	15, b
PMFs						
32	8.41	331.0813	316.0587, 301.0343, 288.0628, 273.0391, 245.0445, 186.0157, 168.0055, 151.0389, 127.0028, 99.0082	C_17_H_14_O_7_	Jaceosidin	14, b
33	9.57	343.1177	328.0940, 313.0705, 285.0756, 270.0516, 257.0804, 242.0575, 211.0756, 199.0237, 181.0130	C_19_H_18_O_6_	5,7,8,4’‐Tetramethoxy‐flavone	20, b
36	10.17	403.1389	388.1155, 373.0919, 355.0803, 327.0861, 301.0704, 258.0531, 229.0341, 183.0289, 165.0550, 127.0385, 98.9440	C_21_H_22_O_8_	Nobiletin	15, a, b
37	10.23	375.1075	360.0840, 345.0605, 327.0499, 317.0659, 302.0422, 274.0468, 215.0186, 197.0084, 183.0297	C_19_H_18_O_8_	Chrysosptertin B	20, b
38	10.58	433.1497	418.1255, 403.1023, 385.0914, 373.0548, 345.0604, 317.0652, 289.0701, 271.0613, 211.0237, 183.0290,	C_22_H_24_O_9_	3,5,6,7,8,3′,4’‐Heptemthoxyflavone	15, a, b
39	10.78	373.1282	358.1045, 343.0812, 328.0578, 315.0853, 297.0757, 283.0802, 271.0600, 229.0322, 211.0237, 193.0131, 183.0289, 151.0383, 135.0441	C_20_H_20_O_7_	Tangeretin	15, a, b
40	11.31	389.1229	374.0991, 359.0758, 341.0653, 331.0808, 316.0576, 285.0874, 215.0185, 197.0083, 163.0752, 148.0519,	C_20_H_20_O_8_	5‐hydroxy‐6,7,8,3′,4′‐pentamethoxyflavone	15, a, b
41	11.89	359.1124	344.0885, 329.0655, 311.0550, 286.0470, 271.0589, 215.0185, 197.0081, 146.0732, 133.0649	C_19_H_18_O_7_	Gardenin B	20, b
Other flavonoids						
14	4.89	289.0707	247.0589, 205.0496, 182.9873, 163.0391, 153.0184, 145.0284, 135.0442, 125.0229, 67.0187	C_15_H_12_O_6_	Eriodictyol	Xia et al. (2019), b
19	5.16	273.0758	255.0649, 231.0656, 207.0656, 171.0289, 153.0183, 147.0441, 129.0185, 119.0494, 91.0547, 67.0186	C_15_H_12_O_5_	Naringenin	15, b
22	5.36	303.0862	285.0761, 219.0655, 201.0547, 177.0548, 171.0289, 153.0184, 117.0338, 89.0392, 67.0186	C_16_H_14_O_6_	Hesperetin	15, b
25	6.57	287.0914	269.0803,245.0806, 214.7453, 203.0698, 179.03387, 171.0288, 161.0598, 153.0183, 133.0649, 79.0548, 67.0186	C_16_H_14_O_5_	Isosakuranetin	14, b
28	7.63	273.0759	207.6625, 189.0546, 171.0292, 153.0184, 147.0442, 123.0442, 119.0495, 107.0494, 91.0548, 67.0184	C_15_H_12_O_5_	Naringenin chalcone	Yoshimura et al. (2009), b
29	8.20	301.0708	286.0473, 258.0524, 229.0502, 179.7487, 153.0181, 118.5655	C_16_H_12_O_6_	Diosmetin	Chen et al. (2019), b
30	8.26	331.0815	316.0581, 301.0344, 288.0634, 273.0395, 255.0288, 245.0446, 175.0403, 151.0393, 131.0495	C_17_H_14_O_7_	Iristectorigenin	14, b
Organic acids						
3	0.44	165.0548	147.0441, 128.3818, 123.0443, 119.0494, 109.0651, 103.0547, 95.0497, 91.0548, 81.0705	C_9_H_8_O_3_	p‐Hydroxy‐cinnamic acid	Ji et al. (2021), b
4	0.44	182.0814	165.0548, 147.0442, 136.0758, 123.0443, 119.0494, 103.0543, 95.0496, 91.0548, 79.5276	C_9_H_11_NO_3_	Tyrosine	3, b
6	0.48	268.1042	240.1231, 234.9566, 230.1042, 222.1126, 210.0374, 206.1177, 199.9880, 195.9935, 194.1177	C_10_H_13_N_5_O_4_	Adenosine	Ji et al. (2021), b
7	0.50	284.0991	257.6237, 209.9237, 171.0495, 152.0569, 135.0303, 110.0352, 85.02880	C_10_H_13_N_5_O_5_	Guanosine	3, b
8	1.52	205.0974	188.0707, 170.0602, 159.0918, 146.0601, 143.0731, 132.0809, 118.0654, 103.0544, 91.0548, 74.0244	C_11_H_12_N_2_O_2_	Tryptophan	3, b
10	3.74	181.0496	163.0390, 145.0285, 129.9791, 111.9687	C_9_H_8_O_4_	Caffeic acid	20, b
12	4.57	195.0654	177.0548, 163.0390, 149.0600, 145.0285, 131.9745, 117.0338, 109.9587, 89.0392	C_10_H_10_O_4_	Isoferulic acid	15, a, b
13	4.88	195.0654	177.0548, 163.0392, 149.0599, 145.0286, 135.0443, 117.0339, 89.0392	C_10_H_10_O_4_	Ferulic acid	15, b
24	6.29	120.0657	102.0554, 84.0450, 75.0448, 74.0608, 69.7176, 65.0394, 56.0503	C_4_H_9_NO_3_	L‐Threonine	Han et al. (2018), b
Alkaloids						
1	0.31	144.1020	84.0814, 71.0498, 65.9154, 58.0659, 55.05502	C_7_H_13_NO_2_	Stachydrine	Pang et al. (2017), a, b
2	0.31	168.1021	150.0915, 135.0680, 132.0810, 121.0650, 119.0495, 107.0494, 93.0704, 91.0548	C_9_H_13_NO_2_	Synephrine	15, a, b
Coumarins						
9	2.36	341.0867	179.0339, 166.4433, 151.0387, 135.0441, 107.0496, 79.0547	C_15_H_16_O_9_	Esculin	16, b
11	4.37	179.0341	155.5042, 137.0599, 135.0442, 123.0444, 107.0496, 95.0497, 79.0549	C_9_H_6_O_4_	Esculetin	17, b
31	8.36	207.0653	192.0419, 179.0698, 164.0469, 151.0755, 148.0519, 139.0755, 121.0651, 118.0416, 103.0544	C_11_H_10_O_4_	5,7‐Dimethoxycoumarin	15, b
Other compounds						
5	0.44	127.0391	109.0288, 99.0445, 81.0341, 71.0499, 57.0343, 53.0394	C_6_H_6_O_3_	5‐Hydroxymethylfurfural	10, b
27	7.12	209.0811	194.0848, 186.0543, 177.0548, 163.0388, 149.0599, 145.0285, 117.0338, 102.9706, 89.0391	C_11_H_12_O_4_	Methyl ferulate	10, b
34	9.70	471.2018	425.1956, 409.2018, 367.1917, 339.1958, 265.1222, 213.0907, 161.0599, 149.0960, 133.0649, 105.0703, 95.0133	C_26_H_30_O_8_	Limonin	14, a, b
35	10.05	223.0967	208.0997, 199.9978, 195.0923, 177.0549, 149.0235, 138.8562, 130.0740, 121.0287, 107.0861	C_12_H_14_O_4_	Ethyl4’‐hydroxy‐3′‐methoxycinnamate	10, b
42	13.67	219.1745	203.1432, 201.1639, 191.1790, 173.1329, 161.1326, 159.1170, 147.1169	C_15_H_22_O	α‐Cyperone	14, b

### 3.3 Identification of seven bioactive components by HPLC–PDA analysis

**FIGURE 2:**
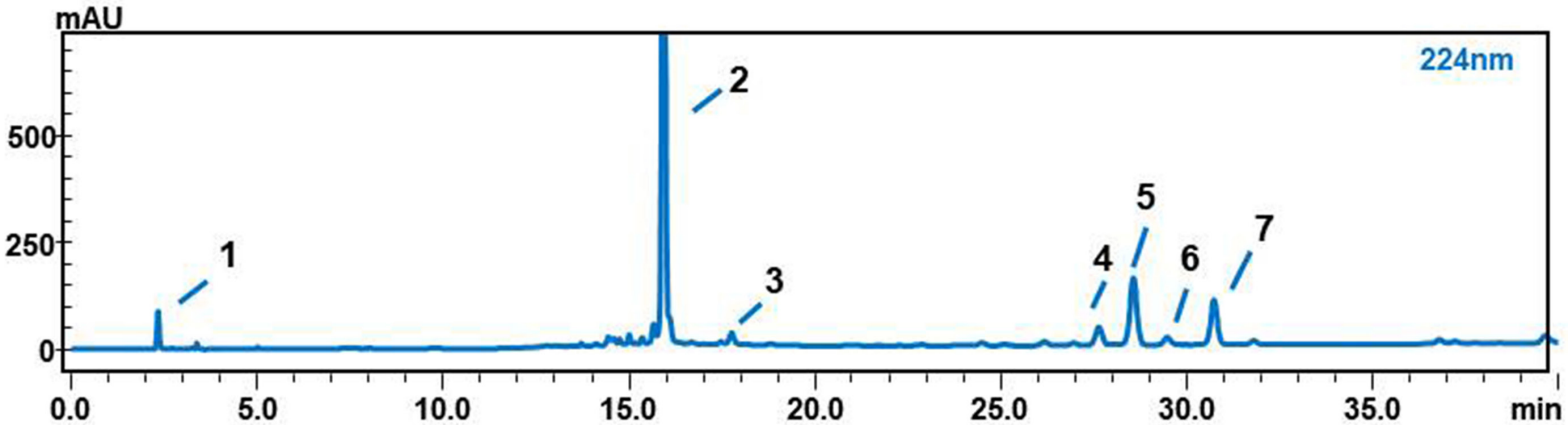
HPLC–PDA chromatograms of GQP. 1. Synephrine 2. Hesperidin 3. Limonin 4. Nobiletin 5. HMF 6. Tangeretin 7. 5‐HPMF.

As shown in Table 3, the linear correlation, repeatability, precision, recovery, and stability of the method were all validated. The results indicated that the method was adapted to quantitatively analyze seven compounds with good linear correlations, whose *R* ^2^ were all greater than 0.99. Moreover, the relative standard deviations (RSDs) of the recovery, repeatability, stability, and precision were wholly less than 3.00%, while the recovery was within the scope of 87.58%–108.71%. The HPLC separation diagram is shown in Figure 2. The above good experimental results can provide a reference for further determination of the content determination method of seven active ingredients in GQP.

**TABLE 3:** Linear correlation, repeatability, precision, stability, and recovery investigation of seven chemical compounds.

Compound	Regression equation	*R* ^2^	Linear range (μg/ml)	LOD (μg/ml)	LOQ (μg/ml)	Repeatability RSD(%)	Precision RSD(%)	Stability RSD(%)	Recovery	Recovery RSD (%)
Synephrine	y = 22,379x‐2072.6	0.9998	5.78–57.8	0.0554	0.1846	1.07	1.86	0.11	96.33	0.66
Limonin	y = 7810.8x + 150,551	0.9980	7.96–39.8	0.0546	0.1819	2.37	2.06	2.87	92.77	1.83
Hesperidin	y = 17,689x + 310,247	0.9991	79.04–790.4	0.0298	0.0993	2.68	2.53	0.33	90.94	1.56
Nobiletin	y = 44,645x + 6729.7	0.9998	12.54–125.4	0.0070	0.0233	0.52	0.49	1.49	103.53	1.88
HMF	y = 31,523x	0.9997	1.56–31.2	0.0173	0.0576	2.23	1.42	2.76	96.24	2.22
Tangeretin	y = 45,142x	0.9998	7.88–78.8	0.0054	0.0181	0.53	0.83	0.51	87.58	2.46
5‐HPMF	y = 33,554x	0.9998	5.36–53.6	0.0113	0.0377	1.54	0.49	0.92	108.71	0.59

As observed in Table 4, GQP has a high concentration of hesperidin (160.78 ± 0.95 mg**·**g^−1^). It can be clearly seen that the content of nobiletin, tangeretin, 5‐HPMF, and HMF in GQP was 4.29 ± 0.02 mg**·**g^−1^, 3.07 ± 0.01 mg**·**g^−1^, 0.51 ± 0.01 mg**·**g^−1^, and 0.18 ± 0.004 mg**·**g^−1^, respectively, suggesting that nobiletin and tangeretin were the primary PMFs in GQP. Besides, GQP was also rich in synephrine (5.57 ± 0.05 mg**·**g^−1^) that is capable of inhibiting the conversion of carbohydrates to lipids (Fu et al., 2020) and limonin (4.11 ± 0.10 mg**·**g^−1^).

**TABLE 4:** The results of determination for seven bioactive components in GQP by HPLC–PDA (mg·g^−1^)^a^.

Sample	Synephrine	Limonin	Hesperidin	Nobiletin	HMF	Tangeretin	5‐HPMF
GQP	5.57 ± 0.05	4.11 ± 0.10	160.78 ± 0.95	4.29 ± 0.02	0.18 ± 0.004	3.07 ± 0.01	0.51 ± 0.01

^a^ Mean ± SD; *n* = 3.

## 4 DISCUSSION AND CONCLUSION

The analysis results of volatile component indicated that the extraction yield of GQP was 1.18%, and the content of volatile components was lower than that of CRP, which may be related to the not completely immature oil chamber in GQP. The relative content of terpenes was the highest, accounting for about 70%, of which γ‐Terpinene had the highest content (33.39%), followed by D‐Limonene (22.95%). And it has been reported that γ‐Terpinene and D‐Limonene possessed wide pharmacological activities, such as antioxidant, anticancer, anti‐inflammatory, and gastroprotective (Wang et al., 2018). On the contrary, D‐Limonene was the most abundant in the volatile oil of CRP, followed by γ‐Terpinene (Zhu et al., 2021), which may be connected with the different recovery times. Compared with the components of CRP, the existing research showed that the difference in volatile components between GQP and CRP primarily depended on the content of D‐Limonene and γ‐Terpinene. It was the γ‐Terpinene in GQP that was the major component of volatile oil and the D‐Limonene was the essential component of CRP (Yi et al., 2015). On the other hand, 42 nonvolatile compounds were identified from the methanol extract of GQP by UHPLC‐Q‐Exactive Orbitrap‐MS, including 23 flavonoids, nine organic acids, two alkaloids, three coumarins, and five other compounds. Among them, 23 flavonoids contained eight flavonoid glycosides, eight PMFs, and seven other flavonoids. Nine compositions were reported for the first time in CRPV, including two flavonoids, one organic acid, three coumarins, and three other compounds, and these compounds were vitexin, iristectorigenin, isoferulic acid, esculin, esculetin, 5,7‐Dimethoxycoumarin, 5‐Hydroxymethylfurfural, methyl ferulate, and ethyl4’‐hydroxy‐3′‐methoxycinnamate. Research indicated that the main nonvolatile components of CRP and GQP were flavonoids, including flavonoid glycosides, polymethoxy flavonoids, and other flavonoids (He et al., 2021; Zheng, Liu, et al., 2020). Seven chemical components of GQP were not found in CRP, including three flavonoids, one organic acid, two coumarins, and one other compound. These compounds were eriodictyol, diosmetin, iristectorigenin, L‐Threonine, esculin, esculetin, and ethyl4’‐hydroxy‐3′‐methoxycinnamate. Furthermore, the result of quantitative analysis for seven bioactive components suggested that GQP was especially abundant in hesperidin (160.78 ± 0.95 mg**·**g^−1^) that was much higher than that of required on Qing pi in Chinese Pharmacopoeia of 2020 edition (not less than 5.0%). The existing preclinical studies and clinical trials have demonstrated that hesperidin possessed therapeutical action in a variety of diseases owing to its lipid‐lowering and insulin‐sensitizing properties (Li & Schluesener, 2017; Parhiz et al., 2015). What is more, GQP was abundant in polymethoxylated flavones as well, mainly nobiletin and Tangeretin (4.29 ± 0.02 mg•g^−1^ and 3.07 ± 0.01 mg•g^−1^, respectively). Recent studies reported that polymethoxylated flavones from citrus could inhibit cancer proliferation, including non‐small‐cell lung cancer, gastric cancer, and so on (Da et al., 2016; Wang et al., 2020). Additionally, the contents of limonin and synephrine were 0.56% and 0.41%, respectively. During the growth period of citrus fruit, the contents of limonin and synephrine first increased, then gradually decreased. Synephrine, as the main alkaloid in citrus, is applied in dietary supplements for weight loss since its potential benefits in increment of fat oxidation (Ruiz‐Moreno et al., 2021). There is evidence that limonin also has a wide variety of bioactivities, including anticancer and anti‐inflammation (Song et al., 2021; Wang et al., 2018).

In this study, GC–MS and UHPLC‐Q‐Exactive Orbitrap‐MS were conducted for the comprehensive analysis of the chemical components of GQP, of which 56 volatile components and 42 nonvolatile components were determined. Further quantitative analysis results showed that GQP was rich in flavonoids, especially hesperidin, nobiletin, and tangeretin. The method established in this study is simple and efficient, which makes a supplement to the research basis of chemical components of GQP. If the quantitative analysis of its main components can be further carried out, it will provide a reference for the selection of quality evaluation index, and provide a data foundation for further pharmacological activity research of GQP.

## CONFLICT OF INTEREST

The authors declare no conflicts of interest.

## ETHICAL APPROVAL

This study does not involve any human or animal testing.

## INFORMED CONSENT

Written informed consent was obtained from all study participants.

## Data Availability

This is an open‐access article under the terms of the Creative Commons Attribution License, which permits use, distribution, and reproduction in any medium, provided the original work is properly cited.
